# Effective Post-Exposure Treatment of Ebola Infection 

**DOI:** 10.1371/journal.ppat.0030002

**Published:** 2007-01-19

**Authors:** Heinz Feldmann, Steven M Jones, Kathleen M Daddario-DiCaprio, Joan B Geisbert, Ute Ströher, Allen Grolla, Mike Bray, Elizabeth A Fritz, Lisa Fernando, Friederike Feldmann, Lisa E Hensley, Thomas W Geisbert

**Affiliations:** 1 Special Pathogens Program, National Microbiology Laboratory, Public Health Agency of Canada, Winnipeg, Manitoba, Canada; 2 Department of Medical Microbiology, University of Manitoba, Winnipeg, Manitoba, Canada; 3 Department of Immunology, University of Manitoba, Winnipeg, Manitoba, Canada; 4 Virology Division, United States Army Medical Research Institute of Infectious Diseases, Fort Detrick, Frederick, Maryland, United States of America; 5 Uniformed Services University of the Health Sciences, Bethesda, Maryland, United States of America; 6 Biodefense Clinical Research Branch, Division of Clinical Research, National Institute of Allergy and Infectious Diseases, National Institutes of Health, Bethesda, Maryland, United States of America; Washington University School of Medicine, United States of America

## Abstract

Ebola viruses are highly lethal human pathogens that have received considerable attention in recent years due to an increasing re-emergence in Central Africa and a potential for use as a biological weapon. There is no vaccine or treatment licensed for human use. In the past, however, important advances have been made in developing preventive vaccines that are protective in animal models. In this regard, we showed that a single injection of a live-attenuated recombinant vesicular stomatitis virus vector expressing the Ebola virus glycoprotein completely protected rodents and nonhuman primates from lethal Ebola challenge. In contrast, progress in developing therapeutic interventions against Ebola virus infections has been much slower and there is clearly an urgent need to develop effective post-exposure strategies to respond to future outbreaks and acts of bioterrorism, as well as to treat laboratory exposures. Here we tested the efficacy of the vesicular stomatitis virus-based Ebola vaccine vector in post-exposure treatment in three relevant animal models. In the guinea pig and mouse models it was possible to protect 50% and 100% of the animals, respectively, following treatment as late as 24 h after lethal challenge. More important, four out of eight rhesus macaques were protected if treated 20 to 30 min following an otherwise uniformly lethal infection. Currently, this approach provides the most effective post-exposure treatment strategy for Ebola infections and is particularly suited for use in accidentally exposed individuals and in the control of secondary transmission during naturally occurring outbreaks or deliberate release.

## Author Summary

Being highly pathogenic for humans and monkeys and the subject of former weapons programs makes Ebola virus one of the most feared pathogens worldwide today. Due to a lack of licensed pre- and post-exposure intervention, our current response depends on rapid diagnostics, proper isolation procedures, and supportive care of case patients. Consequently, the development of more specific countermeasures is of high priority for the preparedness of many nations. In this study, we investigated an attenuated vesicular stomatitis virus expressing the Ebola virus surface glycoprotein, which had previously demonstrated convincing efficacy as a vaccine against Ebola infections in rodents and monkeys, for its potential use in the treatment of an Ebola virus infection. Surprisingly, treatment of guinea pigs and mice as late as 24 h after lethal Ebola virus infection resulted in 50% and 100% survival, respectively. More important, 50% of rhesus macaques (4/8) were protected if treated 20 to 30 min after Ebola virus infection. Currently, this approach provides the most effective treatment strategy for Ebola infections and seems particularly suited for the use in accidental exposures and the control of human-to-human transmission during outbreaks.

## Introduction

*Editor's Note: The potential efficacy of pre- and post-exposure prophylaxis against Ebola virus infection, as well as the fundamentally important question of whether neutralizing antibodies are important for Ebola virus resistance, is addressed by a related manuscript in this issue of* PLoS Pathogens. *Please see doi:10.1371/journal.ppat.0030009 by Oswald et al.*

Infection with the filoviruses, in particular Zaire ebolavirus (ZEBOV), *Sudan ebolavirus,* or Marburg virus (MARV), causes a severe haemorrhagic fever (HF) in humans and nonhuman primates that is often fatal [1–3]. In addition to the sporadic outbreaks that have occurred in humans in Central Africa since 1976 and caused more than 1,800 human infections with a lethality rate ranging from 53% to 90%, Ebola virus (EBOV) has also decimated populations of wild apes in this same region [4]. At this time, there is no preventive vaccine or post-exposure treatment option available for human use.

Much remains to be learned about these highly virulent viruses; however, important advances have been made over the last decade in understanding how filoviruses cause disease and in developing preventive vaccines that are protective in nonhuman primates [1,5]. For example, a recombinant replication-defective adenovirus vaccine completely protected nonhuman primates from uniformly lethal ZEBOV infection [6,7]. More recently, we generated live-attenuated recombinant vesicular stomatitis viruses (VSV) expressing the transmembrane glycoproteins (GP) of ZEBOV (VSVΔG/ZEBOVGP) and MARV (VSVΔG/MARVGP) and the glycoprotein precursor of Lassa virus (VSVΔG/LASVGPC) [8] and showed that these completely protected cynomolgus macaques against lethal challenge with the corresponding filoviruses and arenavirus [9,10]. Progress in developing therapeutic interventions against the filoviruses has been much slower [5]. Limited success was achieved in using an anticoagulant to treat EBOV infections [11], and very recently the VSV-based MARV vaccine platform (VSVΔG/MARVGP) demonstrated astonishing efficacy in post-exposure treatment of MARV-infected macaques [12]. Other than that, no post-exposure modality has been able to protect nonhuman primates against lethal filovirus infections [5,13,14].

There is clearly an urgent need to develop filovirus-specific effective post-exposure strategies to respond to future outbreaks in Central Africa, to counter acts of bioterrorism, and to treat laboratory exposures such as the recent EBOV exposures that occurred in the United States and Russian laboratories [15,16]. Post-exposure vaccine treatment is successful in preventing or modifying viral diseases such as rabies [17,18], hepatitis B [19], and smallpox [20,21] in humans, as well as MARV HF in nonhuman primates [12]. However, the faster disease course and higher lethality of ZEBOV in human and nonhuman primates may limit the success of a similar approach for EBOV HF. Here, we show remarkable efficacy of the VSV-based EBOV vaccine platform in the post-exposure treatment of rodents and nonhuman primates infected with ZEBOV. Currently, this is the most promising post-exposure treatment strategy for EBOV HF and is particularly suited for use in accidentally exposed individuals and in the control of transmission in the event of natural or deliberate outbreaks.

## Methods

### Vaccine Vectors and ZEBOV Challenge Viruses

The recombinant VSV expressing the GPs of ZEBOV (strain Mayinga), MARV (strain Musoke), or Lassa virus (strain Josiah) were generated as described recently using the infectious clone for the VSV, Indiana serotype (kindly provided by J. Rose) [8]. Briefly, the appropriate open reading frames for the GPs (ZEBOV, Mayinga, MARV, Musoke) were generated by PCR, cloned into the VSV genomic vectors lacking the VSV G gene, sequenced, and originally rescued using the method described earlier [8,22]. ZEBOV (strain Kikwit) was isolated from a patient of the EBOV outbreak in Kikwit in 1995 [23]. The mouse- and guinea pig-adapted ZEBOV strains (MA-ZEBOV and GA-ZEBOV, respectively) were generated by serial passages in the different rodent species until uniformly lethal [24,25].

### Animal Studies

#### Rodents.

Female BALB/c mice, 5–6 wk old, were purchased from Charles Rivers (Quebec, Canada). The animals (groups of five) were treated by intraperitoneal (i.p.) inoculation of 2 ×10^5^ plaque forming units (pfu) of VSVΔG/ZEBOVGP into the left and right site of the abdomen (100 μl each). Naïve control animals were immunized with the same volume of Dulbecco's Mimimal Essential Medium (DMEM) by the same route. The mice were challenged i.p. with 1,000 LD_50_ of MA-ZEBOV into the left and right site of the abdomen (100 μl each). Female guinea pigs (Hartley strain), approximately 250 g, were purchased from Charles Rivers (Quebec, Canada). The animals (groups of six) were i.p.-treated with 2 × 10^5^ pfu of VSVΔG/ZEBOVGP into the left and right site of the abdomen (500 μl each). Naïve control animals were immunized with the same volume of DMEM by the same route. The guinea pigs were challenged i.p. with 1,000 LD_50_ of GA-ZEBOV into the left and right site of the abdomen (500 μl each). All rodents (mice and guinea pigs) were weighed daily for a minimum of 11 d following challenge and observed for clinical symptoms according to an approved scoring sheet (ruffled fur, slowing activity, loss of body conditions, labored breathing, hunched posture, bleeding, paralysis). Surviving animals were kept three times longer than the death of the last control animal. All rodent work was performed in the Biosafety Level (BSL)-4 biocontainment facility at the National Microbiology Laboratory of the Public Health Agency of Canada and was approved by the Canadian Science Centre for Human and Health Animal Care Committee following the guidelines of the Canadian Council on Animal Care.

#### Rhesus macaques.

Ten healthy adult Macaca mulatta of Chinese origin (3–6 kg) were used for this study. Briefly, all ten macaques were challenged by intramuscular (i.m.) inoculation with 1,000 pfu of ZEBOV, strain Kikwit. Approximately 20–30 min after ZEBOV challenge, eight of the animals received an i.m. injection with a dose of 2 × 10^7^ pfu of the VSVΔG/ZEBOVGP vector expressing the ZEBOV GP that was divided among four different anatomical locations (right and left triceps and right and left caudal thigh). Two animals served as experimental controls, of which one received an equivalent dose of the VSVΔG/MARVGP vector expressing the MARV GP and the other the VSVΔG/LASVGPC vector expressing the Lassa virus glycoprotein precursor by the same routes. All animals were checked twice daily for clinical symptoms of ZEBOV HF using an established score sheet. Swab samples (oral, nasal, rectal) and blood were taken prior to ZEBOV challenge and on days 3, 6, and 10 post ZEBOV challenge. Survivors were kept for more than 50 d. All nonhuman primate studies were performed in BSL-4 biocontainment at United States Army Medical Research Institute of Infectious Diseases (USAMRIID) and were approved by the USAMRIID Laboratory Animal Care and Use Committee. Animal research was conducted in compliance with the Animal Welfare Act and other Federal statues and regulations relating to animals; experiments involving animals adhere to the principles stated in the *Guide for the Care and Use of Laboratory Animals,* National Research Council, 1996. The facility used is fully accredited by the Association for Assessment and Accreditation of Laboratory Animal Care International.

### Haematology and Serum Biochemistry

Total white blood cell counts, lymphocyte counts, red blood cell counts, platelet counts, haematocrit values, total haemoglobin, mean cell volume, mean corpuscular volume, and mean corpuscular haemoglobin concentration were determined from nonhuman primate blood samples collected in tubes containing EDTA, by using a laser-based haematology analyzer (Beckman Coulter, http://www.beckmancoulter.com). The white blood cell differentials were performed manually on Wright-stained blood smears.

### Virus Detection

RNA was isolated from nonhuman primate whole blood and swabs using appropriate RNA isolation kits (Qiagen, http://www1.qiagen.com). ZEBOV RNA was detected using primer pairs targeting the L genes [ZEBOV: RT-PCR, nt position 13344–13622; nested PCR, nt position 13397–13590]. The sensitivity of the ZEBOV-specific RT-PCR is approximately 0.1 pfu/ml. ZEBOV titration was performed by plaque assay on Vero E6 cells from all blood and selected organ (adrenal, ovary, lymph nodes, liver, spleen, pancreas, lung, heart, brain) and swab samples [23]. Briefly, increasing 10-fold dilutions of the samples were adsorbed to Vero E6 monolayers in duplicate wells (0.2 ml per well); thus, the limit for detection was 25 pfu/ml.

### Immune Responses

IgG and IgM antibodies against ZEBOV were detected with an enzyme-linked immunosorbent assay (ELISA) using purified virus particles as an antigen source [6]. Neutralization assays were performed by measuring plaque reduction in a constant virus:serum dilution format as previously described [9,26]. Briefly, a standard amount of ZEBOV (∼100 pfu) was incubated with serial 2-fold dilutions of the serum sample for 60 min. The mixture was used to inoculate Vero E6 cells for 60 min. Cells were overlayed with an agar medium, incubated for 8 d, and plaques were counted 48 h after neutral red staining. End point titres were determined by the dilution of serum, which neutralized 50% of the plaques (PRNT_50_).

### Cellular Immune Responses

Peripheral blood mononuclear cells were isolated from rhesus macaque whole blood samples by separation over a Ficoll gradient. Approximately 1 × 10^6^ cells were stained for cell surface markers, granzyme B, and viral antigen using monoclonal antibodies. Staining procedures were performed as previously described [27].

## Results

**Figure 1:**
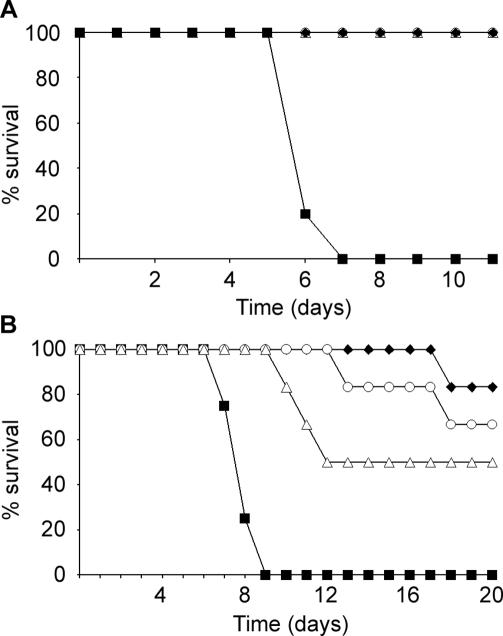
Kaplan-Meier Survival Curves for Mice and Guinea Pigs Given Post-Exposure Treatment for ZEBOV Infection (A) Mice (groups of five animals) were infected with 1,000 LD_50_ of MA-ZEBOV by i.p. injection. At various times points 24 h prior to challenge (○), 30 min after challenge (♦), or 24 h after challenge (▵) they were treated with  2 ×  10^5^ pfu of VSVΔG/ZEBOVGP by i.p. injection. The controls (▪) were left untreated and all died. All treated animals survived the challenge. (B) Guinea pigs (groups of six animals) were infected with 1,000 LD_50_ of GA-ZEBOV by i.p. injection. At various times points 24 h prior to challenge (○), 1 h after challenge (♦), or 24 h after challenge (▵) they were treated with 2 × 10^5^ pfu of VSVΔG/ZEBOVGP by i.p. injection. The controls (▪) were left untreated and all died.

To test the concept that the VSVΔG/ZEBOVGP vaccine may have utility as a post-exposure treatment for EBOV HF, we investigated its efficacy in two rodent models, mouse [25] and guinea pig [24], and a rhesus macaque model [11]. Initially, we treated groups of five BALB/c mice with i.p. injections of 2 × 10^5^ pfu of the VSVΔG/ZEBOVGP vaccine 24 h prior to challenge or 30 min or 24 h post i.p. challenge with a 1,000 LD_50_ of the mouse-adapted ZEBOV (MA-ZEBOV) [25]. The immunization dose chosen was relatively high considering that as little as 2 × 10^0^ pfu still conferred complete protection against the same challenge dose (unpublished data). Animals were weighed every day and scored for clinical symptoms (see Methods). Untreated control animals (naïve controls) rapidly lost weight, developed severe clinical symptoms, and died on day 6 post-challenge (Figures 1A and S1A). Surprisingly, all treated mice survived independent of the time of treatment (Figure 1A). Those animals treated 24 h prior to challenge did not show any clinical symptoms, whereas animals treated post-challenge developed mild clinical symptoms. With all protected groups, mild weight loss was observed during the first day post-challenge (Figure S1) indicating virus replication prior to clearance and survival.

Next, we treated three groups of guinea pigs (Hartley strain; six animals per group) with i.p. injection of 2 ×10^5^ pfu of the VSVΔG/ZEBOVGP either 24 h before challenge or 1 or 24 h after challenge with 1,000 LD_50_ of the guinea pig-adapted ZEBOV (GA-ZEBOV) [24]. Disease progression was followed and measured as described for the mice. Untreated guinea pigs (naïve controls) showed weight loss at day 5 post-challenge progressing to death on days 7 to 9 (Figures 1B and S1B). Unlike the mice, the treatment groups were not fully protected (Figures 1 and S1). Two animals (33%) died from the group treated 24 h prior to challenge; one (17%) and three (50%) animals died from the groups treated 1 and 24 h post-challenge, respectively (Figures 1B and S1B). In all cases, the development of clinical symptoms, weight loss and time to death, were significantly delayed. All surviving animals lost weight and became sick with a degree of severity that correlated very well with disease outcome. The final survival rates were 66% for the pre-treatment group (24 h prior to challenge) and 83% and 50% in the 1- and 24-h post-treatment groups, respectively (Figures 1B and S1B).

**Figure 2:**
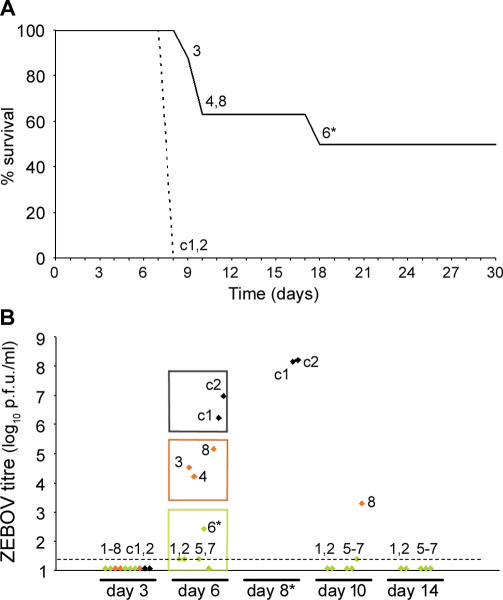
Survival and Plasma Viraemia for Rhesus Monkeys Given Post-Exposure Treatment for ZEBOV Infection (A) Kaplan-Meier survival curves for animals treated with ∼2 ×10^7^ pfu of VSVΔG/ZEBOVGP (subjects 1 to 8, solid line) or VSV control vectors (subjects c1 and c2, dotted line) 20–30 min after i.m. challenge with 1,000 pfu of ZEBOV. (B) Plasma viraemia of animals treated with VSVΔG/ZEBOVGP or VSV control vectors 20–30 min after i.m. challenge with 1,000 pfu of ZEBOV. Viraemia was determined by plaque assay at indicated time points. The asterisk indicates that on day 8 post-challenge viraemia levels were only determined for the control animals (subjects c1 and c2). Plasma viraemia levels at day 6 post-ZEBOV challenge could be separated into three different groups. Control animals, which received VSV control vectors (black square), developed high plasma viraemias (>6 log_10_ pfu/ml). Animals treated with VSVΔG/ZEBOVGP, which developed fulminant EBOV HF and succumbed to ZEBOV challenge (orange square), developed moderate plasma viraemias (∼4–6 log_10_ pfu/ml), while animals treated with VSVΔG/ZEBOVGP, which survived (green square), had low plasma viraemias (≤1.4 log_10_ pfu/ml). Subject 6 did not develop fulminant disease consistent with EBOV HF and succumbed on day 18 from a secondary bacterial infection.

Encouraged by the success in the rodent models, we treated eight rhesus monkeys (subjects 1 to 8) with i.m. injections of the VSVΔG/ZEBOVGP vaccine (2 × 10^7^ pfu), and two rhesus monkeys (subjects c1 and c2) with VSV control vaccines (2 × 10^7^ pfu) (see Methods) 20 to 30 min after challenge with 1,000 pfu of ZEBOV. The immunization and challenge doses were equivalent to what had been used in previous successful pre-exposure vaccine studies [6,9]. All animals became febrile by day 6 and haematology data indicated evidence of illness by day 6, usually manifested as lymphopenia, in most of these animals (Table 1). Surprisingly, 50% of the VSVΔG/ZEBOVGP-treated animals (subjects 1, 2, 5, and 7) survived the lethal ZEBOV challenge (Figure 2A; Table 1) without showing signs of severe disease, while three VSVΔG/ZEBOVGP-treated macaques (subjects 3, 4, and 8) developed characteristic ZEBOV HF including fever, perturbations in clinical chemistry values, and macular rashes (Figure S2); these animals died on days 9 (subject 3) and 10 (subjects 4 and 8) (Figure 2A; Table 1). Notably, all VSVΔG/ZEBOVGP-treated animals that succumbed to the ZEBOV challenge (subjects 3, 4, and 8) developed plasma viraemia on day 6 between 1 × 10^4^ and 1 × 10^6^ pfu/ml, whereas plasma viraemia was transient in the animals that survived (subjects 1, 2, 5, and 7) and did not exceed 1 × 10^2^ pfu/ml on day 6 (Figure 2B). The final VSVΔG/ZEBOVGP-treated macaque (subject 6) died on day 18 (Figure 2A; Table 1). This animal had a transient low-level ZEBOV viraemia on day 6 and had cleared the ZEBOV infection by day 10 (Figure 2B). Furthermore, the animal never developed clinical symptoms consistent with severe ZEBOV HF, and organ infectivity titration showed no evidence of infectious ZEBOV in any of the tissues surveyed at post-mortem. Pathology results showed that this macaque died from disseminated septicaemia and peritonitis caused by Streptococcus pneumoniae as demonstrated by immunohistochemistry (unpublished data). The source of the bacterial infection is unknown. Both monkeys treated with the VSV control vectors (subjects c1 and c2) developed severe symptoms over the disease course with plasma viraemia titres in excess of 1 × 10^6^ pfu/ml on day 6, macular rash (Figure S2) evident by day 7, and death on day 8 after ZEBOV challenge (Figure 2A; Table 1) with peak viraemia titre of >1 × 10^8^ pfu/ml (Figure 2B). In addition, all animals were also tested for VSV viraemia using RT-PCR (unpublished data). In accordance with our previous results [9,10], VSV RNA was detected in most immunized animals only at day 3 post-immunization indicating transient viraemia of the vaccine vector. There was no correlation between VSV viraemia and survival.

**Table 1:**
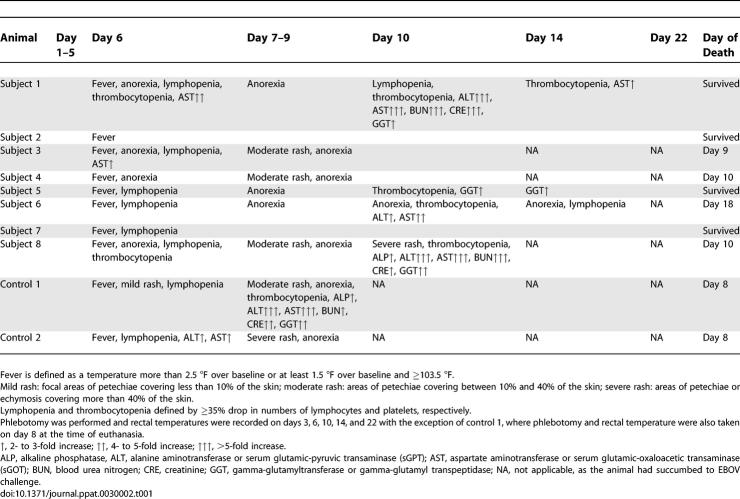
Clinical Findings

**Figure 3:**
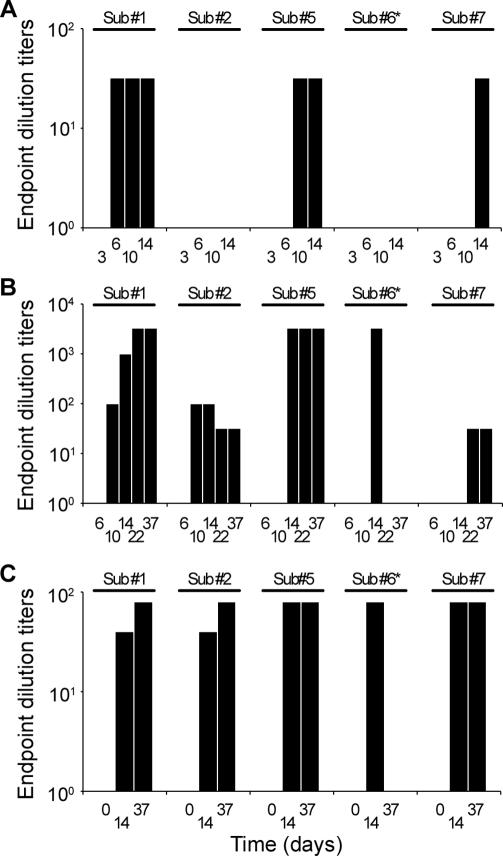
Serological Response Profile for Rhesus Monkeys Given Post-Exposure Treatment for ZEBOV Infection IgM (A), IgG (B), and development of EBOV-neutralizing antibodies (C) in sera of animals treated with 2 × 10^7^ pfu of VSVΔG/ZEBOVGP 20–30 min after i.m. challenge with 1,000 pfu of ZEBOV.

All four animals that survived the ZEBOV challenge (subjects 1, 2, 5, and 7), and the animal that survived until day 18 (subject 6), developed ZEBOV-specific humoral immune responses with low titre IgM antibodies detected on days 6–14 (subjects 1, 5, and 7) (Figure 3A) and moderate IgG antibody titres detected on days 10–22 (subjects 1, 2, 5, 6, and 7) (Figure 3B). Neutralizing antibody titres to ZEBOV (1:80) were detected on days 14–37 after challenge in all four animals that survived the ZEBOV challenge (subjects 1, 2, 5, and 7) and the animal that survived until day 18 (subject 6) (Figure 3C). Humoral immune responses could not be detected in any of the non-survivors although these animals lived until day 9 and 10 post-challenge, which was sufficient to mount detectable IgM and IgG responses in the surviving animals.

We also evaluated changes in populations of peripheral blood mononuclear cells during the course of the study to identify any differences between the rhesus monkeys treated with the VSVΔG/ZEBOVGP vector and the controls. A rapid loss of CD4^+^ lymphocytes, CD8^+^ lymphocytes, and NK cells has been reported during ZEBOV infection of nonhuman primates [28]. In this study, we also detected a decline in the circulating CD4^+ ^and CD8^+^ (2%–10% decrease) lymphocyte populations on day 6 in most of the animals regardless of treatment or outcome with a 7%–22% decrease and 2%–10% decrease in cell numbers observed, respectively (Table 1). However, the percentage of NK cells did not drop in any of the animals treated with VSVΔG/ZEBOVGP vector on day 6, but markedly increased. Interestingly, a sharp decline in NK cell number (10% decrease) was observed on day 10 in one of the animals treated with the VSVΔG/ZEBOVGP vector. Similarly, a marked increase in B cells was noted for all animals regardless of treatment or outcome on day 6, followed by a decline in B cell number on day 10.

## Discussion

Although no EBOV vaccine is currently licensed for human use, recent advances have been made and efficacy studies in nonhuman primates with several platforms have been encouraging [6,7,9]. Far less progress has been made in developing treatment interventions for EBOV infections [5,13,14]. Thus, there is clearly a need to develop effective strategies to respond to future EBOV outbreaks in Africa and to counter acts of bioterrorism using EBOV. Additionally, the potential EBOV exposure involving a researcher at a United States Army laboratory [16] and the unfortunate death of a Russian scientist after an accidental exposure to EBOV [15], underscore the need for medical countermeasures for post-exposure prophylaxis. Recently, a post-exposure strategy to mitigate the coagulation disorders that typify filoviral infections improved survival from 0% to 33% in the rhesus macaque model of ZEBOV HF [11]. Here, we show a significant advance in treating EBOV infections.

Our data clearly demonstrate the efficacy of the VSV-based EBOV vaccine vector in post-exposure treatment in three relevant animal models. In the mouse model it was possible to protect all animals following challenge with treatment as late as 24 h post infection. It is known from previous data that treatments and vaccines given to mice are more effective than seen in guinea pigs and nonhuman primates [1,2,13]. However, in this case it was possible to protect over 50% of guinea pigs and 50% of nonhuman primates from uniformly lethal ZEBOV challenge. It should be noted that mice received about 10 or 100 times more vaccine per weight than guinea pigs and nonhuman primates, respectively. Thus, it is possible that further optimization of dosing strategies could improve the results.

The rhesus macaques that survived infection all controlled the virus within the first 6 d of infection. The data clearly show that moderate or high-level viraemia on day 6 invariably resulted in a fatal outcome (Figure 2). In the current study, we can conclude that neutralizing antibodies were not essential for infection control (Figure 3) since they were not detected until after the animals had cleared the EBOV infection. Circulating CD4^+^ and CD8^+^ T cells were reduced in number in all animals regardless of treatment (Table 1); this indicates that the initial control of infection may not require classical T-cell responses. The time course for EBOV HF in rhesus macaques is very short (∼8 d) and therefore, CD8^+^ cytotoxic T-cell responses are very unlikely to be involved in the control of the infection because the cell numbers of specific responding cells could not have peaked until after the infection was controlled. The primary immune correlate of protection seems to be the rapid development of non-neutralizing antibody that was only seen in the protected animals (Figure 3). This, coupled with the NK-cell increase in the VSVΔG/ZEBOVGP-treated animals, may have resulted in significantly enhanced killing of virus-infected primary target cells and, consequently, elimination of the ZEBOV infection. An important role of NK cells for protection has also been described for immunization with virus-like particles [29].

Clearly, the adaptive response is essential to promote survival as animals immunized with the control VSV-based vaccines succumbed to the ZEBOV challenge (Figure 2, Table 1). Both control animals died on day 8, which is the historical mean for rhesus monkeys infected by the same route and dose with this seed stock (historical *n* = 23). However, other mechanisms probably contribute as well. Recently, Noble and colleagues described a new paradigm for an interfering vaccine in which one of the antiviral mechanisms of action is intracellular interference with the replication of the lethal wild-type virus [30]. In the current study, the VSV vectors exploit the EBOV GP, which largely determines host cell tropism and mediates viral entry [31]. We have demonstrated that the VSV vectors expressing the ZEBOV GP will infect the same cells as wild-type ZEBOV in vitro [8]. Also, the VSVΔG/ZEBOVGP vectors replicate significantly faster than wild-type ZEBOV [8]. Therefore, it is possible that these vectors compete with ZEBOV through viral interference. Clearly, even mild to moderate inhibition of ZEBOV replication may delay the course of infection and tip the balance in the favor of the host.

VSV has been shown to be a potent inducer of the innate and adaptive immune system [32–34]. In contrast, EBOV has acquired mechanisms to counteract the innate immune responses of the host at different levels [1,2,35]. The virion protein (VP) 35 of ZEBOV functions as an inhibitor of type I interferon production by blocking the activation of IRF-3 [36–38]. In addition to VP35, the ZEBOV protein VP24 functions as an inhibitor of type I interferon signaling by blocking nuclear accumulation of activated STAT-1 [35,39]. Recently, it was suggested that VP24 blocks the downstream signaling cascades activated by type I interferon by inhibiting the phosphorylation of p38 [40]. Therefore, treatment with the VSV vectors might induce or boost the innate immune response in the host, and thus, counteract the immune inhibitory effect of EBOV. In this case, the host will mount a nonspecific innate immune response allowing for time to develop a specific adaptive response that can overcome the EBOV infection and again tip the balance in favor of survival of the host.

In a historical context, it is important to note that the mechanism for post-exposure protection of humans against smallpox and rabies are also not fully understood. For post-exposure treatment of rabies, levels of neutralizing antibodies have been used as a measure of protection. However, several studies of HIV-infected patients with likely or proven exposure to rabies showed that these patients failed to develop neutralizing antibodies after post-exposure rabies vaccination, yet there were no reports of death of these patients attributed to rabies [41,42]. Moreover, studies in mice suggest that cell-mediated immunity may play an essential role in post-exposure protection [43]. In the case of smallpox, post-exposure protection is presumed to be due in part to differences in the route of exposure and growth kinetics of the wild-type variola virus versus the vaccinia vaccine [20]. Briefly, infection with variola usually starts in the upper and lower respiratory tract with subsequent spread to lymphoid tissues. Thus, the natural variola infection proceeds much slower than post-exposure i.m. vaccinia vaccination, which bypasses the respiratory tract infection. In addition, it appears that vaccinia has a shorter incubation period than variola virus resulting in a more rapid development of cell-mediated immunity and neutralizing antibody. However, a recent study using monkeypox in the macaque model demonstrated better results with antiviral therapy than post-exposure vaccination [44].

Post-exposure treatment with the VSV-based MARV vaccine vector against MARV challenge was more potent and resulted in complete survival, no disease, and undetectable viraemia [12]. The development of symptoms and viraemia in MARV-infected rhesus monkeys is delayed compared with ZEBOV [12,45], which may explain the difference in efficacy in post-exposure treatment with the VSV-based vectors. The efficacy of the VSV-based EBOV vector in post-exposure treatment might be increased by a higher treatment dose or multiple treatments over a longer period of time as is being done in post-exposure treatment of rabies [46]. Alternatively, combination therapy should be considered to increase therapeutic efficacy. In the case of EBOV, post-exposure treatment with the VSV-based EBOV vector could be combined with the previously published post-exposure strategy to mitigate the coagulation disorders [11].

Nevertheless, the VSV-based ZEBOV vaccine currently provides the most effective and promising single treatment strategy for EBOV HF. It is likely that the mechanism of protection by the VSV-based vaccine is multifactorial; while NK cells and antibody responses appear to be important to survival, viral interference and innate immune response are almost certainly essential in delaying the progression of the ZEBOV infection and extending the window for the adaptive response to become functional.

Post-exposure treatment is particularly suited for use in accidentally exposed individuals and in the control of secondary transmission during naturally occurring outbreaks or deliberate releases. Our results also suggest that this VSV platform might be even more beneficial as a fast-acting single-shot preventive vaccine. Finally, this system also provides an excellent opportunity to study the fundamental mechanisms that lead to such devastating disease following infection with ZEBOV.

## Supporting Information

Figure S1 Weight Loss of Mice and Guinea Pigs Given Post-Exposure Treatment for ZEBOV Infection
(A) Mice (groups of five animals) were infected with 1,000 LD_50_ of MA-ZEBOV by i.p. injection. At various times points 24 h prior to challenge (○), 30 min after challenge (♦), or 24 h after challenge (▵) they were treated with 2 ×10^5^ pfu of VSVΔG/ZEBOVGP by i.p. injection. The controls (▪) were left untreated and all died. All treated animals survived the challenge.
(B) Guinea pigs (groups of six animals) were infected with 1,000 LD_50_ of GA-ZEBOV by i.p. injection. At various times points 24 h prior to challenge (○), 1 h after challenge (♦), or 24 h after challenge (▵) they were treated with 2 ×10^5^ pfu of VSVΔG/ZEBOVGP by i.p. injection. The controls (▪) were left untreated and all died. †, animals that succumbed to infection in the treated groups.
(980 KB TIF)

Figure S2 Clinical Symptoms
Macular rash covering the inguinal region and inner leg of a VSVΔG/ZEBOVGP-treated macaque (subject 8) that succumbed 10 d after ZEBOV challenge.
(5.1 MB TIF)

### Accession Numbers

The GenBank (http://www.ncbi.nlm.nih.gov/Genbank) accession number for the ZEBOV Mayinga strain is AF272001; the accession number for the MARV Musoke strain is Z12132.

## Abbreviations

EBOV: Ebola virus
GP: glycoprotein
HF: haemorrhagic fever
i.m.: intramuscular
i.p.: intraperitoneal
MARV: Marburg virus
pfu: plaque forming unit
VP: virion protein
VSV: vesicular stomatitis virus
ZEBO: *Zaire ebolavirus*
